# Reliability and validity of self-assessment of mouth opening: a validation study

**DOI:** 10.1186/1472-6831-12-48

**Published:** 2012-11-12

**Authors:** Daniel Satnam Singh Saund, David Pearson, Thomas Dietrich

**Affiliations:** 1 Specialist Registrar Oral Surgeon, University of Birmingham, St Chads Queensway, Birmingham, B4 6NN, UK; 2 The School of Dentistry, University of Birmingham, St Chad’s Queensway, Birmingham, B4 6NN, UK

**Keywords:** Reproducibility, Validity, Measurement, Scale, TheraBite

## Abstract

**Background:**

The objective of this study was to evaluate the reproducibility and validity of patients’ mouth opening measurements in a research setting.

**Methods:**

Firstly, 68 patients made repeated self-measurements of mouth opening using a cardboard scale (Therabite Range of Motion Scale – TRMS). Secondly, 80 patients enrolled in a clinical trial on morbidity after lower third molar surgery, made daily assessments during the postoperative week. Patients’ measurements were then compared to gold-standard clinicians’ measurements.

**Results:**

Reliability of patients’ measurements was excellent with an intraclass correlation coefficient of 0.92. The patient’s measurements correlated well with the gold-standard clinician’s measurements, both for the first 68 patients (Pearson’s rho ranging from 0.86 to 0.90, p < 0.0001) as well as for the 80 patients enrolled in the clinical trial (rho = 0.82, p < 0.0001 at day 2, rho = 0.83, p < 0.0001 at final visit).

**Conclusions:**

TRMS can be used by patients to produce reproducible and valid mouth opening measurements.

## Background

Trismus is a common complication following surgical and other procedures performed within the oral cavity and its associated musculature, including third molar surgery [1,2]. In addition, it is also a complication of disease processes affecting the temporomandibular joint and the dentition. Many studies involve monitoring the improvement in patient’s mouth opening as a measure of postoperative morbidity [2] or as an outcome measure following therapeutic interventions [3]. Assessment of trismus in a clinical setting is straightforward; however, more frequent recordings of trismus, in particular during postoperative healing, may be necessary in clinical research. In an outpatient setting, this would make a reproducible and valid method of patient self-assessment of mouth opening highly desirable.

The TheraBite® range of motion scale™ is a simple and inexpensive cardboard scale designed for self-assessment of trismus for patients with TMJ disorders. However, its reproducibility and validity has not been assessed.

The aim of this study was to evaluate the reproducibility and validity of patients’ self assessment of mouth opening with the TheraBite® range of motion scale™.

## Method

The study had local NHS Research & Development approval, was approved by the South Staffordshire Local Research Ethics Committee and was conducted according to the guidelines outlined in the Helsinki Declaration. The present study was conducted in two separate, consecutive phases. In the first phase, conducted as part of an undergraduate student research elective project, the acceptability, reproducibility and validity of the scale was evaluated in a convenience sample of 68 patients attending Birmingham Dental Hospital for outpatient consultation clinics in the Oral Surgery department. Patients had been referred by General Dental Practitioners for consultations regarding TMJ problems or for third molar removal. After providing informed consent, patients were instructed in the use of the scale. A pilot study involving the first 13 patients revealed that careful instructions and practice with the clinician was needed for the patient to comfortably and accurately use the device. Based on these initial findings, an illustrated step-by-step guide was developed that was given to patients (available from the authors upon request). In addition to this, instructions on how to use the scale included practicing its use in front of a mirror.

Following this pilot phase, 55 consecutive patients were asked to make 4 repeated measurements of their mouth opening with the scale. For this purpose, they were provided with 4 scales and envelopes. In order to avoid bias by patients remembering their previous measurements, they were asked to place each scale in a provided corresponding envelope and seal it immediately after use. In addition, the numerical scale was masked using a marker pen. Patients were asked to leave at least 5 minutes between measurements. The fourth and final measurement was done in the presence of the investigator. The investigator noted the patients’ ability to locate the cardboard notch accurately on the lower incisors and to accurately position the scale on the upper incisors.

In order to assess the validity of the patients’ readings, the investigator measured a patient’s interincisal distance using a steel ruler both before and after the patient’s measurements. Finally, patients were then asked to record on a 5-point Likert scale how strongly they agreed or disagreed with the statements “I found this device easy to use” and “I would feel confident to record my mouth opening with this device every day over a week if needed”.

Following the evaluation of the use of the scale in this first phase, we used the scale in an ongoing clinical trial on third molar morbidity (NCT01145820). We present results for the first 80 patients enrolled in this study. Briefly, patients 18–65 years old of both genders, who required the surgical removal of a single impacted lower third molar where enrolled. Exclusion criteria included long-term anti-inflammatory medications, regular vitamin or mineral supplementation, pregnancy or lactation. Immediately following surgical removal of a single impacted lower third molar, patients were instructed in the use of the scale and asked to measure their mouth opening once during the evening of each postoperative day. The first reading was done on the day of surgery and then on each day during the postoperative week. Patients attended for follow-up appointments 2 days and 7 days after surgery.

A baseline recording of the interincisal distance was taken by the operating surgeon using a steel ruler immediately pre-operatively. This recording was repeated by the surgeon on post operative days 2 and 7 when the patient was asked to return to clinic for review. A diary of the patient’s daily recordings was also collected on day 7 at their final review appointment.

### Statistical analysis

Summary statistics were calculated as appropriate. For the first phase of this study, paired t-tests were used to assess differences between the first and last measurements by patients and clinicians. To assess reproducibility of patients’ measurements, we calculated the intraclass correlation coefficient [4]. Finally, to assess validity, the Pearson correlation coefficient was calculated comparing the first patient to the first clinician measurement, the final patient to the final clinician measurement, and finally, the mean of all patient measurements and the mean of all clinical measurements. For phase 2 of this study, summary statistics were calculated as appropriate. Pearson correlation coefficients were calculated to assess the validity of the patients’ measurements compared to the corresponding clinical measurements. These correlations were made between the clinician’s and patient’s measurements on day 2 and the clinician’s measurement at the final visit and the patient’s measurement recorded on the previous evening. All statistical tests were two-sided at α = 0.05 using a statistical software (STATA 11, Stata Corp, College Station, TX, USA).

## Results

### Phase 1

A total of 68 patients were enrolled. The first 13 patients were part of a pilot phase where problems with patient instructions and practice were identified and addressed. Problems identified included a tendency for patients to place the cardboard notch in the interdental space between the lower incisors. Furthermore, patients tended to bend the cardboard when positioning the device against the upper incisors. In response to these findings, a step-by-step guide was developed to aid patient instruction and practice was performed in front of a mirror. Thus, patient measurements from the first 13 patients were disregarded for analyses and a total of 55 patients (20 male, 35 female, mean age 38 ± 15 years) were included in the final sample. When comparing the first and final clinician measurement for all 68 patients there was a slight increase in mean mouth opening from 45.0 (range: 12–67) mm to 46.0 (range: 13 – 72) mm (p = 0.01). For the 55 patients included in the final sample, the difference was smaller and not statistically significant (45.4 mm and 45.9 mm, p = 0.18). The mean of the interincisal distance as measured by these patients increased from 45.3 mm at the first measurement to 47.1 mm at the final measurement (p = 0.004). The intraclass correlation coefficient was 0.92 (95% CI 0.88, 0.95), indicating excellent reproducibility [4]. Finally, the patients’ measurements correlated well with the gold-standard clinicians’ measurement, with Pearson’s correlation coefficients ranging from 0.86 to 0.90.

According to the attending clinician, 94% of patients were able to correctly position the cardboard notch on the lower incisors and 86% were able to accurately position the scale on the maxillary incisors. 98% of patients agreed or strongly agreed with the statement that the scale was easy to use and 92% indicated that they would be happy to use it over a week at home if needed.

### Phase 2

**Figure 1:**
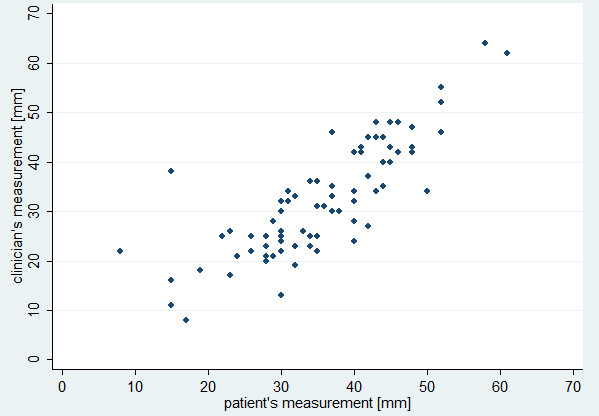
Scatterplot of clinician’s measurement and patient’s measurement on day 2 (rho = 0.82, p < 0.0001).

**Figure 2:**
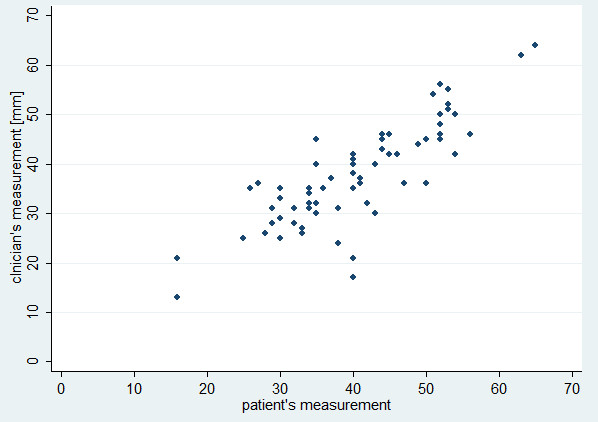
Scatterplot of clinician’s vs. patient’s final measurement (rho = 0.83, p < 0.0001).

We included 80 patients (44 [55%] females, 36 [45%] males) with a mean age of 29.5 (SD 7.9) years. The mean preoperative interincisal distance as measured by the clinician was 47.8 (SD 7.8) mm. This was reduced to 31.9 (SD 11.6) mm on the second postoperative day and 38.4 (SD 10.2) mm at the final visit. The mean interincisal distance as measured by the patient on the evening of the second postoperative day was 35.5 (SD 10.2) mm, and 40.8 (10.1) mm on the evening preceding the final visit. Patients’ and clinicians’ measurements were highly correlated (rho = 0.82, p < 0.0001 at day 2 [Figure 1], rho = 0.83, p < 0.0001 at final visit [Figure 2]).

All patients recorded their measurements in the diaries and, although this was not formally assessed, no problems were identified by the patients with regards to the use of the scale.

## Discussion

Our study has shown excellent reproducibility and validity of patients’ self-assessment of mouth opening using a cardboard scale, both within a hospital setting and when used by patients at home in their personal environments. Furthermore, the vast majority of patients found the scale easy to use and suitable for use in a clinical research setting. The study also demonstrated that careful patient instruction is necessary including demonstration and practice measurements with the help of a mirror.

To our knowledge, this is the first study assessing the reproducibility and validity of patients’ self-assessment of mouth-opening. Based on the results of this study, we feel that the scale is a simple, safe and inexpensive tool that is suitable for use in clinical research studies, where review appointments for clinician measurements are either not feasible or where more frequent assessment in between clinician assessments is deemed desirable. Hoole et al. have previously evaluated the reliability and validity of the scale for measurements taken by nurses and had mixed results [5]. It is difficult to reconcile any differences as these authors used different statistical measures of agreement.

Our study has several limitations. Firstly, 68 patients enrolled in the first phase of the study made all assessments in a relatively short time period in a hospital setting, rather than repeated daily assessments over several days or weeks more likely to be used in a clinical research application. This may have resulted in an overestimate of reliability due to patients not being blinded to their previous assessment; however, we aimed to minimize such bias by blocking out the scale numbers and asking patients to seal each scale in an envelope immediately after use. Secondly, in the first phase, clinicians’ measurements indicated that the patients were able to open slightly wider at the end of the study, suggesting a ‘training effect’ which was mirrored in the patients’ measurements. This increase in (gold-standard) mouth-opening would have lead to an underestimate of the intraclass correlation coefficient, which assumes a ‘constant’ gold-standard. Furthermore, the majority of patients had normal mouth-opening and it is possible that reproducibility is different in patients with severely limited mouth-opening. A previous study found no differences in reproducibility of clinician measurements between patients with or without trismus [6].

Thirdly, it must be noted that during the second phase of the study, patients recorded their mouth opening measurements the night before or after the gold standard readings were taken by the clinician. This may explain, at least partially, the slightly lower correlation between clinicians’ and patients’ measurements in this phase compared to the first phase of the study.

Furthermore, the scale can only be used in patients who have intact incisor teeth, a limitation shared with any method measuring interincisal distance. The method may therefore be of limited use in some patients or in some research scenarios (e.g., oncology).

A strength of this study is that the study population comprised of patients in oral surgery consultation and TMJ clinics, as well as surgical postoperative patients which represent the target population for use of the scale in a clinical and/or research scenario.

## Conclusions

This study has demonstrated that a simple cardboard scale can provide reliable and valid assessment of interincisal distance by patients and may be a valuable research tool in a setting where patients are not reviewed postoperatively. It has the distinct advantage of being cheap, and easily provided for use by patients without being overly cumbersome. This study has shown that patients are more than able to use the scale and achieve accurate results when compared to gold standard clinician measurements.

## Abbreviations

NHS: National Health Service; TMJ: Temporomandibular joint; TRMS: Therabite Range of Motion Scale.

## Competing interests

The scales were provided free of charge by Platon Medical Ltd., Eastbourne, East Sussex, UK.

## Authors’ contributions

DS contributed to the design of the study, data collection and drafted the manuscript. DP contributed to the design of the study and data collection. TD contributed to the design of the study, data collection and performed data analysis. All authors read and approved the final manuscript.

## Financial support

This study was funded by departmental funds. The scales were provided free of charge by Platon Medical Ltd., Eastbourne, East Sussex, UK.

## Pre-publication history

The pre-publication history for this paper can be accessed here:

http://www.biomedcentral.com/1472-6831/12/48/prepub
